# Effects of an exercise and hypocaloric healthy eating intervention on indices of psychological health status, hypothalamic-pituitary-adrenal axis regulation and immune function after early-stage breast cancer: a randomised controlled trial

**DOI:** 10.1186/bcr3643

**Published:** 2014-04-14

**Authors:** John M Saxton, Emma J Scott, Amanda J Daley, M Nicola Woodroofe, Nanette Mutrie, Helen Crank, Hilary J Powers, Robert E Coleman

**Affiliations:** 1 School of Rehabilitation Sciences, Faculty of Medicine and Health Sciences, Room 2–8 Queen’s Building, University of East Anglia, Norwich NR4 7TJ, United Kingdom; 2 Warwick Medical School, University of Warwick, Coventry, UK; 3 Department of Primary Care Clinical Sciences, University of Birmingham, Birmingham, UK; 4 Biomedical Research Centre, Sheffield Hallam University, Sheffield, UK; 5 Institute for Sport, Physical Education and Health Sciences, University of Edinburgh, Edinburgh, UK; 6 Centre for Sport and Exercise Science, Sheffield Hallam University, Sheffield, UK; 7 Human Nutrition Unit, Department of Oncology, University of Sheffield, Sheffield, UK; 8 CR-UK/YCR Sheffield Cancer Research Centre, Weston Park Hospital, University of Sheffield, Sheffield, UK

## Abstract

**Introduction:**

Many women experience emotional distress, depression and anxiety after a diagnosis of breast cancer. Psychological stress and depression have been associated with hypothalamic-pituitary-adrenal (HPA) axis dysregulation that may adversely affect immune system functioning and impact upon survival. This study investigated the effects of a lifestyle intervention on indices of psychological health status, HPA axis regulation and immune function in overweight women recovering from early-stage breast cancer treatment.

**Methods:**

A total of 85 women treated for breast cancer 3 to 18 months previously were randomly allocated to a 6-month exercise and hypocaloric healthy eating program plus usual care or usual care alone (control group). Women in the intervention group received three supervised exercise sessions per week and individualized dietary advice, supplemented by weekly nutrition seminars. Depressive symptoms (Beck Depression Inventory version II: BDI-II), perceived stress (Perceived Stress Scale: PSS), salivary diurnal cortisol rhythms; inflammatory cytokines (IL-6 and Tumor necrosis factor-α), leukocyte phenotype counts, natural killer (NK) cell cytotoxicity and lymphocyte proliferation following mitogenic stimulation were assessed at baseline and 6-month follow up.

**Results:**

Compared with the control group, the intervention group exhibited a reduction in depressive symptoms (adjusted mean difference, 95% confidence intervals (95% CI): −3.12, −1.03 to −5.26; *P* = 0.004) at the 6-month follow-up but no significant decrease in PSS scores (−2.07, −4.96 to 0.82; *P* = 0.16). The lifestyle intervention also had a significant impact on diurnal salivary cortisol rhythm compared with usual care alone, as evidenced by an increase in morning salivary cortisol at the 6-month follow-up (*P* <0.04), indicating a change in HPA axis regulation. Women in the control group had higher total leukocyte, neutrophil and lymphocyte counts in comparison to the intervention group at the 6-month follow-up (*P* ≤0.05), whereas there was no difference in NK cell counts (*P* = 0.46), NK cell cytotoxicity (*P* = 0.85) or lymphocyte proliferation responses (*P* = 0.11) between the two groups.

**Conclusion:**

Our results show that the lifestyle intervention resulted in a reduction in depressive symptoms and a normalisation of HPA axis regulation. Such changes could have important implications for long-term survival in women recovering from early-breast cancer treatment.

**Trial registration:**

Current Controlled Trials: ISRCTN08045231

## Introduction

Many women experience emotional distress, depression and anxiety after a diagnosis of breast cancer, which can persist for prolonged periods, irrespective of the clinical treatment outcome [1,2]. Studies show that a quarter of breast cancer patients have clinically important levels of emotional distress up to 12 months after treatment [3] and almost 50% of women experience depression and/or anxiety during this period [1]. Psychological stress and depression have been associated with hypothalamic-pituitary-adrenal (HPA) axis dysregulation in breast cancer survivors, including aberrations in diurnal cortisol rhythm [4,5], and elevated levels of the inflammatory cytokine IL-6 [6] that may impair immune system functioning [7] and adversely impact upon survival [8,9].

The importance of healthy lifestyle behaviours to prevent the adverse effects of weight gain after a breast cancer diagnosis is widely acknowledged and sustainable lifestyle change was recently identified as one of the key areas for future research [10]. Lifestyle interventions that incorporate exercise and healthy eating advice could also have an important role in modulating indices of psychological health status during the early recovery phase after breast cancer treatment. Physical inactivity and poor dietary habits are independently associated with depressive symptoms in breast cancer survivors [11,12], whereas evidence suggests that regular exercise [13] and positive dietary change [14] can improve depressive symptoms and psychological wellbeing. By improving psychological health status, lifestyle interventions have the potential to impact upon the HPA axis regulation and enhance immune function in women recovering from breast cancer treatment. This could improve long-term outcome, as restoration of immune function after treatment is predictive of survival [15,16].

We recently reported the results of a randomised controlled trial that investigated the effects of an exercise and hypocaloric healthy eating intervention on body weight and metabolic biomarkers associated with disease recurrence in women recovering from early-stage breast cancer treatment [17]. Women in the intervention group experienced a wide range of health benefits, including reductions in central adiposity, dietary fat intake, total cholesterol and resting diastolic blood pressure and an improvement in cardiopulmonary fitness [17]. Here, in the same patient cohort, we report the effects of the lifestyle intervention on indices of psychological health status, HPA axis regulation and immune function.

## Methods

### Participant recruitment

This study recruited 90 overweight women with a body mass index (BMI) >25 kg/m^2^, who had completed surgery, chemotherapy and radiotherapy for early-stage breast cancer (stage I to III) 3 to 18 months previously. Biological samples were unavailable for five women in the sample population, leaving an evaluable sample size of 85 women. Patients receiving adjuvant endocrine treatments were eligible, and those yet to complete a one-year course of adjuvant trastuzumab were also included, subject to acceptable cardiac function determined by a multi-gated acquisition (MUGA) scan and consultant approval. Exclusion criteria included: concomitant hormone replacement therapy (HRT) or oral contraceptives; metastatic or active loco-regional disease; physical or psychiatric impairment limiting physical mobility; severe nausea, anorexia or other conditions precluding participation in exercise, the consumption of alternative/complementary diets or use of high-dose antioxidant supplements; and those already engaged in regular exercise. Patients were recruited from the Cancer Clinical Trials Centre at Weston Park Hospital, Sheffield UK or through local cancer support services, the local media or word of mouth. Ethics approval was obtained from the South Sheffield Research Ethics Committee and all participants provided written informed consent prior to the first assessment visit.

### Sample size

Change in body weight was chosen as the primary outcome variable for calculation of sample size [17]. Using the data of Utter *et al*. [18], we estimated that recruitment of 90 women (45 in each group) would give us 90% power to detect a clinically meaningful reduction in body weight between the groups at the two-sided α level of 0.05. In addition, using Beck Depression Inventory II (BDI-II) data from our previous exercise trial [13], a sample size of 38 patients per group gave us 80% power to detect a change in BDI of 6 points at the alpha level of 0.05.

### Randomisation and allocation concealment

Following the assessment of outcome variables at baseline, patients were randomly allocated to one of two groups: (i) lifestyle intervention (n = 44), or (ii) control group (n = 41). The control group received a healthy eating booklet, *Eat Well* (Food Standards Agency, UK), which also included brief advice on keeping active. Minimisation was used to balance the potentially confounding variables of chemotherapy and treatment with tamoxifen, aromatase inhibitors or no hormone therapy. Randomisation was performed by an independent researcher at the Clinical Trials Research Unit, University of Leeds. The randomisation sequence was not disclosed until patients had completed their baseline assessments.

### Lifestyle intervention

Details of the pragmatic lifestyle intervention were published previously [17]. Briefly, the 24-week lifestyle intervention combined three supervised exercise sessions each week with an individually tailored hypocaloric healthy eating programme. Exercise sessions (including one to three women) comprised 30 minutes of aerobic exercise (65 to 85% age-predicted maximum heart rate) using one or more of a treadmill, cross-trainer, cycle ergometer and/or rowing ergometer, followed by 10 to 15 minutes of muscle strengthening exercises using resistance bands, hand weights and stability balls. Each participant also received one-to-one individualized dietary advice and written information (*Weight Loss On A Plate*, Scottish Dietetic Association). The written information included guidance on portion sizes for common foods in each food group and a healthy eating plan. The goal was to reduce the patient’s total daily calorie intake to 600 kcal below their calculated energy requirements, thereby inducing an estimated steady weight loss of up to 0.5 kg each week. Additional weekly small-group nutrition education seminars included topics such as dietary fat intake, hydration, achieving a healthy balanced diet and alcohol consumption. In the control group, contact with researchers was limited to assessment sessions. Participants in the control group were offered three exercise sessions at the university exercise research facility and general exercise and dietary advice after the final follow-up.

### Outcome measures

Outcomes were assessed at baseline (pre-randomisation) and at 24 weeks post randomisation in both groups. Questionnaire outcomes (depressive symptoms and perceived stress) were assessed by a trained technician who was blinded to the group allocation. Saliva and blood analytes were measured blindly by members of the research team.

### Depressive symptoms and perceived stress

Depressive symptoms were assessed using the BDI, Version II^©^ (BDI-II) [19], which has a range of 0 to 63, with each item rated on a 4-point Likert-type scale ranging from 0 to 3. Perceived stress was assessed using the perceived stress scale (PSS) [20]. The scale was developed to measure the extent to which respondents appraise situations in their life to be stressful during the past month. The 14-item scale has a range of 0 to 56, with items rated on a Likert-type scale ranging from 0 to 4, and with higher scores indicating higher levels of perceived stress.

### Saliva and blood analysis

Saliva samples were collected using salivettes® (Sarstedt, Leicester, UK) containing a piece of absorbance gauze, at 8 am, 12 noon, 5 pm and 9 pm over three consecutive days and stored at 4°C until collection. Saliva samples from each daily time point were pooled and mixed, before being cleared by centrifugation at 300 *g* for 10 minutes and stored for later analysis. Blood (15 to 20 mL) was drawn from an antecubital vein between 8 and 10 am following a 12-hour overnight fast. Saliva samples for analysis of diurnal cortisol rhythms and serum samples for analysis of inflammatory cytokines (IL-6 and TNF-α) were stored at −80°C until analysis, with duplicate baseline and post-intervention samples analysed in the same batch. Analysis of lymphocyte phenotype and function (T cell/natural killer (NK) cell phenotyping, lymphocyte proliferation in response to phytohemagglutinin (PHA) stimulation and NK cell cytotoxicity) were commenced within 2 hours of blood collection and analysed in duplicate or triplicate. Full blood-count analyses were undertaken in the Haematology Department of the Sheffield Teaching Hospitals NHS Foundation Trust UK. All other analyses were undertaken in the Biomedical Research Centre Laboratory at Sheffield Hallam University UK, as described below.

### HPA axis modulation

Diurnal salivary cortisol concentrations were determined using a high sensitivity ELISA kit (Salimetrics, Newmarket, UK). The detection limit is 0.003 μg/dL and the intra- and inter-assay coefficients of variation were 3.5% and 5.1%, respectively. Salivary cortisol typically shows a diurnal response, being higher in the morning and lower in the evening. Hence, the area under the diurnal salivary cortisol curve (AUC) was calculated using the trapezoidal rule. The inflammatory cytokines, IL-6 and TNF-α, were measured using high-sensitivity ELISA kits (R&D Systems, Oxon, UK). The inter- and intra-assay precision was 7.8% and 7.4%, respectively for IL-6 and 8.4% and 5.3%, respectively for TNF-α. The lower limits of detection were 0.039 ρg/mL and 0.106 ρg/mL, respectively.

### Lymphocyte phenotyping

Absolute counts of helper/inducer (CD3^+^CD4^+^) and suppressor/cytotoxic (CD3^+^CD8^+^) T lymphocytes and NK cells (CD3^−^CD16^+^CD56^+^) were determined using a three-colour whole-blood staining technique (TriTest™ BD Biosciences, Oxon, UK). Briefly, whole blood (100 μl) was incubated for 30 minutes at 4°C with 10 μl of TriTest™ solution (three-colour direct immunofluorescence reagent) in TruCount™ tubes containing a predefined number of lyophilized latex beads (BD Biosciences, Oxon, UK), before the erythrocytes were lysed and the sample analysed immediately using flow cytometry (FAC-Sort flow cytometer with CELLQuest Pro data acquisition and analysis software; BD Biosciences, Oxon, UK).

### NK cell cytotoxicity

Details of the NK cell cytotoxicity assay have been published previously [21]. Briefly, K562 cells (NK cell-sensitive human erythroleukemic cell line) were grown in RPMI 1640 (Gibco-BRL, UK), supplemented with 100 μg/mL streptomycin, 100 U/mL penicillin and 10% fetal bovine serum (complete RPMI: cRPMI), before being labelled with the green fluorescent 3,3′-dioctadecyloxacarbocyanine perchlorate (DIOC18_(3)_, Sigma-Aldrich Ltd., Dorset, UK) at a concentration of 3 mM in dimethyl sulfoxide (DMSO). Unfractionated peripheral blood mononuclear cells (PBMCs) were used as the effector cell population. Peripheral blood was diluted 1:1 with 0.9% w/v NaCl and two volumes of the diluted sample were layered over one volume of Nycoprep 1.077 (Axis-Shield Diagnostics Ltd., Huntingdon, UK). The gradient mixture was centrifuged at 300 *g* for 30 minutes and the PBMCs harvested from the interface. PBMCs were then resuspended in cRPMI (5 × 10^6^ cells/mL) prior to their inclusion as effector cells in the NK cell cytotoxicity assay. PBMC effector cells and DIOC18_(3)_-labelled K562 target cells were mixed together at five effector:target (E:T) cell ratios: 50:1, 25:1, 12.5:1, 6.25:1 and 3.12:1, mixed thoroughly, pelleted by centrifugation at 300 g for 1 minute and incubated for 3 hours at 37°C in 5% CO_2_/95% air in a humid environment. The DNA stain propidium iodide (PI; 10 μg/mL), used to label non-viable cells, was then added immediately prior to flow cytometry analysis for determination of the proportion of live and dead K562 target cells at each E:T cell ratio.

### Lymphocyte proliferation assay

Lymphocyte proliferation was measured using bromodeoxyuridine (BrdU) incorporation during DNA synthesis [22] after stimulation with the non-specific mitogen PHA. PBMCs (50 μl of a 5 × 10^6^ cells/mL suspension in cRPMI) were added to the wells of a 96-well flat-bottom tissue microtitre plate before mixing with 50 μl of PHA at concentrations of 0 μg/mL (culture medium only), 0.1, 0.5, 1.0 and 10.0 μg/mL in cRPMI (final volume of 100 μl per well). Cultures (in triplicate) were incubated at 37°C with 5% CO_2_ for 48 hours and proliferation was assessed using a colorimetric BrdU immunoassay (BrdU Cell Proliferation ELISA: Roche Diagnostics GmbH, Mannheim, Germany). The mean optical density of antigen-stimulated cultures/mean optical density of medium-only cultures was determined for each culture treatment and recorded.

### Data analysis

Intention-to-treat analysis was used to compare patients in the groups to which they were randomly assigned, with missing data being imputed using the SPSS linear interpolation procedure. Three women were lost to follow up in each group. The Shapiro Wilk test was used to check the normality of the data prior to data analysis, with non-normally distributed variables log-transformed to normality before analysis. Comparisons between the groups were analysed using analysis of covariance (ANCOVA), with baseline values used as the covariate. Repeated measures ANOVA was used to assess for changes from baseline within the groups. Unless otherwise stated, normally-distributed data are presented as mean ± SD and non-normally distributed data as median (semi-interquartile range (SIQR)). Categorical data were analysed using the chi-squared test (χ^2^). Statistical significance throughout was taken at the two-sided 5% level (*P* <0.05). All data were analysed using SPSS v19.0 (IBM, Somers, NY, USA).

## Results

The two groups were generally well-matched at baseline for key demographic and anthropometric variables (Table 1). In addition, levels of depression and perceived stress were well-balanced across the two groups. Nearly all the women were white Caucasian and on average, were 7 to 9 months post-treatment. Compliance to the supervised exercise sessions was excellent, with women attending on average 84% (42 to 98%) of the sessions that were offered to them.

**Table 1:** Baseline characteristics of the two groups

**Characteristic**	**Intervention group (n = 44)**	**Control group (n = 41)**
Age, years	55.8 (10.0)	55.3 (8.8)
Body mass, kg	78.1 (10.1)	82.9 (17.0)
Body mass index, kg/m^2^	29.7 (3.5)	31.1 (5.7)
Waist circumference, cm	91.1 (9.9)	94.5 (13.8)
Waist:hip ratio	0.82 (0.07)	0.83 (0.06)
Months post treatment	9.0 (5.5)	7.1 (4.4)
Lymphoedema, n (%)	9 (20.4)	15 (36.6)
Ethnicity		
White, n (%)	43 (98)	41 (100)
Marital status		
Married/cohabitating, n (%)	29 (66)	29 (71)
Single/windowed/divorced, n (%)	15 (34)	12 (29)
Smokers, n (%)	3 (6.8)	1 (2.4)
Hormone treatment		
Chemotherapy, n (%)	26 (59.1)	22 (53.7)
Radiotherapy, n (%)	38 (86.4)	33 (80.5)
Tamoxifen, n (%)	21 (47.7)	21 (51.2)
Arimidex, n (%)	14 (31.8)	10 (24.4)
Herceptin, n (%)	3 (6.8)	5 (12.2)

n, number.

Depressive symptoms were reduced in the intervention group in comparison with controls at the 6-month follow-up (*P* = 0.004). This was due to a greater reduction in BDI-II scores from baseline in the intervention group (*P* <0.001) in comparison with the controls (*P* = 0.02; Table 2). A total of 15 (34%) of participants in the intervention group were at least mildly depressed at baseline (BDI-II ≥14 [19]), in comparison with 2 participants (4.5%) at the 6-month follow-up, whereas in the control group the number of at least mildly depressed participants remained constant (22%) throughout the study period (χ^2^ = 5.93; *P* = 0.02). In contrast, the reduction in PSS score in the intervention group versus controls at the 6-month follow-up was not significant (*P* = 0.16), despite a significant reduction from baseline PSS score (*P* <0.001, Table 2).

**Table 2:** BDI-II and PSS scores at baseline and 6 months in the lifestyle intervention and usual care control groups

	**Intervention group**			**Control group**		
	**Baseline**	**Follow up**	**Δ**	**Baseline**	**Follow up**	**Δ**
BDI-II	11.3 (7.6)	5.1 (4.9)	−6.1 (6.9)**	10.2 (5.5)	7.9 (6.0)	−2.3 (5.8)*
PSS	22.9 (7.2)	18.2 (7.7)	−4.8 (7.9)**	21.5 (5.6)	19.5 (6.8)	−1.9 (6.8)

BDI-II, Beck depression inventory-II; PSS, perceived stress scale; Δ, change from the baseline score; *P <0.05; **P <0.01 in comparison with baseline (within groups).

**Figure 1:**
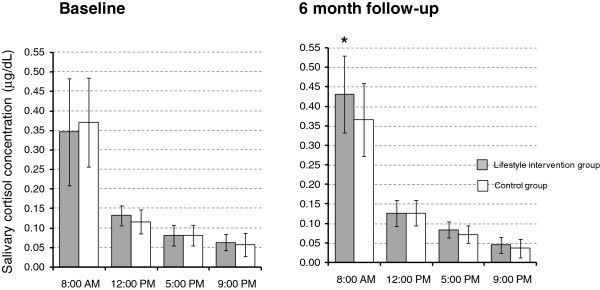
**Diurnal salivary cortisol rhythm at baseline and 6-month follow-up.** Values are medians with error bars representing semi-interquartile range (SIQR). *P = 0.03 for area under the curve (AUC) between groups.

**Figure 2:**
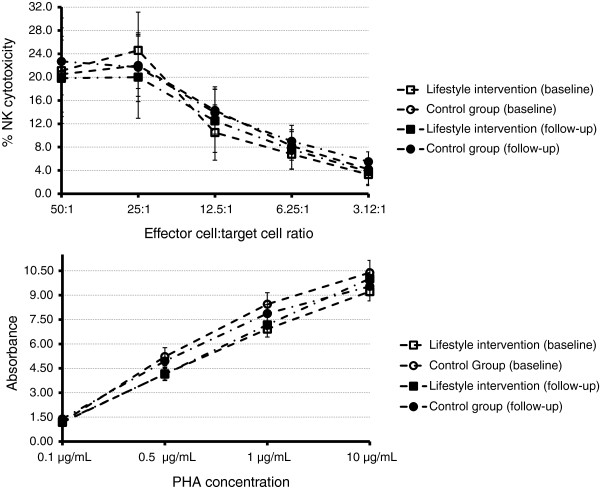
**Natural killer (NK) cell cytotoxicity and lymphocyte proliferation at baseline and 6-month follow-up.** Top, NK cell cytotoxicity at different effector: target cell ratios. Values are medians, with error bars representing semi-interquartile range (SIQR). Bottom, lLymphocyte proliferation responses. Values are means with error bars representing standard error of the mean. PHA, phytohemagglutinin.

A pronounced diurnal cortisol rhythm was evident at baseline in both groups, with highest levels being recorded in the morning sample and lowest values in the evening (Figure 1). Although a similar diurnal cortisol rhythm was apparent in both groups at the 6-month follow-up, an increase in diurnal salivary cortisol rhythm AUC was observed in the lifestyle intervention group in comparison with the controls (*P* = 0.03; Figure 2). This was attributable to a higher post-intervention morning salivary cortisol concentration (*P* = 0.03; Figure 1). In contrast, the lifestyle intervention had no effect on circulating levels of the inflammatory cytokines, IL-6 (*P* = 0.93) or TNF-α (*P* = 0.61; Table 2).

An increase in total leukocyte count in the controls in comparison with the intervention group was observed at the 6-month follow-up (*P* = 0.04; Table 3), attributable to an increase in lymphocyte count (*P =* 0.04) and neutrophil count (*P =* 0.05). The CD3^+^CD4^+^ and CD3^+^CD8^+^ T cell counts showed a different pattern of response between the two groups (*P* ≤0.05; Table 3). However, the lifestyle intervention had no effect on CD4^+^:CD8^+^ T cell ratio (*P* = 0.87), NK cell count (*P* = 0.46), NK cell cytotoxicity (*P* = 0.85) or lymphocyte proliferation in response to PHA stimulation (*P =* 0.11; Table 3 and Figure 2).

**Table 3:** Leukocyte counts and inflammatory cytokines at baseline and follow up

	**Intervention group**		**Control group**		***P*-value**
	**Baseline**	**Follow up**	**Baseline**	**Follow up**	
Leukocyte counts					
Total leukocyte count (10^3^ cells/μl)	5.145 (1.417)	5.156 (1.337)	5.184 (1.237)	5.594 (1.370)	**0.04**
Neutrophil count (10^3^ cells/μl)	3.014 (1.119)	2.958 (1.135)	3.131 (0.912)	3.377 (1.061)	0.05
Monoctye count (10^3^ cells/μl)	0.400 (0.370, 0.430)	0.400 0.360, 0.440	0.400 (0.350, 0.450)	0.400 (0.350, 0.450)	0.63
Lymphocyte count (10^3^ cells/μl)	1.507 (0.414)	1.523 (0.389)	1.446 (0.497)	1.612 (0.449)	**0.04**
CD3^+^CD4^+^T cells (10^3^cells/μl)	0.748 (0.596, 0.900)	0.682 (0.605, 0.759)	0.659 (0.532, 0.786)	0.763 (0.648, 0.878)	**0.02**
CD3^+^CD8^+^ T cells (10^3^cells/μl)	0.474 (0.365, 0.583)	0.402 (0.334, 0.470)	0.360 (0.214, 0.506)	0.409 (0.322, 0.496)	0.05
CD4^+^:CD8^+^ ratio	1.62 (1.36, 1.88)	1.69 (1.40, 1.98)	1.93 (1.51, 2.35)	1.91 (1.49, 2.33)	0.87
NK cells (10^3^ cells/μl)	0.200 (0.175, 0.225)	0.206 (0.177, 0.235)	0.176 (0.147, 0.205)	0.174 (0.144, 0.292)	0.46
Inflammatory cytokines					
IL-6 (ρg/mL)	1.599 (1.259, 1.906)	1.692 (1.377, 2.007)	1.755 (1.456, 2.054)	1.942 (1.602, 2.282)	0.93
TNF-α(ρg/mL)	0.889 (0.779, 0.999)	0.916 (0.767, 1.065)	1.058 (0.895, 1.221)	0.992 (0.870, 1.114)	0.61

Data are presented as means (SD) or medians (semi-interquartile range). *P*-value shown for between-group comparisons. *P*-values in boldface significant at alpha 0.05. NK, natural killer.

## Discussion

Although pragmatic lifestyle interventions could be important for helping to prevent the adverse effects of weight gain after breast cancer treatment [10], few studies have assessed the impact of combined exercise and healthy eating programmes on psychological wellbeing and associated prognostic biomarkers in breast cancer survivors [23,24]. This is the first study to report the effects of a combined exercise and healthy eating intervention on psychological health status, indices of HPA axis regulation and immune function in overweight women during the early recovery phase (3 to 18 months) after stage-I to -III breast cancer treatment. There was excellent compliance with the exercise sessions (84%) and low attrition, with only three participants from each group being lost to follow up in this analysis of psychological and neuro-immunological variables. An improvement in depressive symptoms in the intervention group was not accompanied by a significant reduction in perceived stress. However, the lifestyle intervention also had a significant impact on diurnal salivary cortisol rhythm, as evidenced by an increase in morning salivary cortisol at the 6-month follow-up.

At baseline, BDI-II depression scores in our patient cohort were consistent with previous studies of breast cancer survivors [2,11,25]. Furthermore, the reduction in mean BDI-II depression score from baseline in the intervention group was similar to that previously reported following exercise interventions [25]. Improvements in depression scores have also been observed after lifestyle interventions involving a dietary component to promote healthy eating and healthy body weight maintenance [26,27] but this has not been a consistent finding [23]. Using the criterion of 0.5 SD for our sample population at baseline [28], a reduction in BDI-II score >3 points is clinically meaningful. Hence, the 6.1-point reduction from baseline and between-group difference of >3 points at the 6-month follow-up suggests that women in the intervention group experienced a clinically important change in depressive symptoms. Depressive symptoms in women recovering from breast cancer treatment have been associated with fatigue, pain, sleep disturbances, low libido, poor financial status and social support, and a range of other psychological factors [2]. Physical inactivity and poor dietary habits have also been independently associated with depressive symptoms in breast cancer survivors [11,12].

Evidence suggests that lifestyle interventions, which include support for exercise and dietary behaviour change, may improve depressive symptoms by improving mood, enhancing self-efficacy expectations, reducing intrusive thoughts about breast cancer and future morbidity/mortality and buffering self-concept perceptions [27,29]. Reductions in body weight and/or body fat and improvements in cardiopulmonary fitness (as observed in the intervention participants [17]) could also have a positive impact on depression scores [30,31]. In addition, biological mechanisms, including the release of monoamine neurotransmitters (that is, serotonin, dopamine and norepinephrine) and endorphins during exercise have been proposed to account for improvements in depressive symptoms in physically active individuals [32]. However, it is also important to be mindful of attention effects on depression symptoms, as an improvement in BDI-II score was previously reported in the attention control arm of a short-term exercise trial in a similar cohort of early-stage breast cancer patients [13]. The increased level of personal contact between the intervention group and researchers is a potential limitation of the present study, which needs to be taken into consideration. In summary, continued research is necessary to understand the extent to which such factors influenced the improvement in depressive symptoms in the present study and the impact of changes on long-term prognosis.

Although the intervention group also exhibited a significant reduction in PSS scores from baseline levels, the smaller difference between groups at the 6-month follow-up was not significant. PSS scores in our patient cohort at baseline were comparable to those reported previously for breast cancer survivors [33-37]. However, the lack of effect on this outcome measure contrasts with previously reported reductions (in the range of 4 to 9 points) following stress management and yoga interventions in early-stage breast cancer patients [33-36]. A large proportion of breast cancer survivors are reported to use complementary therapies (including exercise) to manage stress [38], but only one previous study has reported reductions in PSS scale scores following exercise training [37]. The reduction in PSS scores after a 10-week programme of aerobic and resistance exercise was only marginally greater in this study than the non-significant difference between groups in our patient cohort at the 6-month follow-up. Hence, the lack of a significant effect on perceived stress in the present study may be a reflection of low statistical power for this outcome.

The intervention had a significant impact on HPA axis regulation, as evidenced by an increase in diurnal salivary cortisol rhythm at the 6-month follow-up. This was attributable to a higher morning salivary cortisol concentration. Cortisol levels follow a strong circadian rhythm in healthy adults, with the highest levels being evident in the morning after awakening and the nadir occurring at around midnight [39]. A pronounced diurnal cortisol rhythm was apparent at baseline in our patient cohort, and this is consistent with previous studies of non-metastatic breast cancer patients and survivors [4,5]. However, this is the first study to report a steepening of diurnal cortisol rhythm following a lifestyle intervention in breast cancer patients in the early recovery phase after treatment. The implications of this are difficult to define but flatter diurnal cortisol rhythms (blunted morning and/or elevated evening levels) have been associated with depressive symptoms, poor sleep quality and persistent fatigue after breast cancer treatment [5,40]. Hence, the intervention may have influenced morning cortisol levels via its positive impact on one or all of these factors, or via its impact on other factors not monitored in this study. Interestingly, an increase in morning cortisol level, accompanied by improvements in depressive symptoms and psychological stress, was reported after an 8-week stress reduction programme involving yoga, meditation and group discussions in breast cancer patients [33]. This was interpreted as evidence that the intervention resulted in a normalisation of the cortisol response. In a similar way, our results may indicate a modest improvement in HPA axis regulation in the intervention group but further research is needed to confirm this and delineate the precise determinants of HPA axis modulation.

Circulating levels of the inflammatory cytokines IL-6 and TNF-α have also been used as evidence of aberrant HPA axis regulation in breast cancer patients and other populations [6,7,9]. Depression and psychological stress are characterised by elevated levels of IL-6 and TNF-α, [6,41] and IL-6 stimulates cortisol secretion by adrenocortical cells [42]. However, in the present study, levels of these inflammatory cytokines were unchanged at the 6-month follow-up, and this is consistent with previous lifestyle intervention studies involving breast cancer survivors [23,24]. Elevated levels of these inflammatory cytokines have more generally been associated with severe and/or chronic depression and more marked HPA axis dysregulation in previous studies [6,9]. The pronounced diurnal cortisol rhythm at baseline in our patient cohort, coupled with evidence of mild depressive symptoms, suggests a more modest disruption of HPA axis regulation, which probably explains why the intervention had no effect on these inflammatory cytokines.

This is also consistent with our results for immune function. Previous studies show that high levels of stress and depression, coupled with the persistent activation of the HPA axis, impair immune responses and potentially contribute to the development and progression of cancer (reviewed by Reiche *et al*. [7]). However, indices of immune function (lymphocyte proliferation and NK cell cytotoxicity) were unchanged from baseline levels in the present study. Furthermore, the different pattern of response in CD3^+^CD4^+^ and CD3^+^CD8^+^ T cell counts is difficult to interpret, as baseline values were higher in the intervention group. Nevertheless, an increase in total leukocyte count, attributable to an increase in lymphocyte and neutrophil counts, was observed in the controls. The clinical implications of the latter are unknown but surgically treated early-stage breast cancer patients who experienced disease recurrence exhibited higher lymphocyte and neutrophil counts relative to disease-free patients in the 17 months prior to recurrence detection [8]. This contrasts with earlier evidence of an association between low lymphocyte counts and breast cancer recurrence [43]. Although changes in circulating leukocyte counts during recovery from cancer treatment may be indicative of specific and/or non-specific immune reactions to sub-clinical disease [8,43], the clinical significance of such changes in our patient cohort is difficult to interpret without the longer-term follow-up of clinical endpoints such as disease-free interval and mortality.

## Conclusions

In conclusion, this study shows for the first time that an improvement in depressive symptoms resulting from supervised exercise and healthy eating advice in the early recovery phase, 3 to 18 months, after breast cancer treatment is accompanied by a significant change in diurnal salivary cortisol rhythm. The increase in morning salivary cortisol at the 6-month follow-up suggests a normalisation of HPA axis regulation, which may be linked to the improvement in psychological health status. The precise determinants and clinical significance of these changes warrants further investigation.

## Abbreviations

ANCOVA: analysis of covariance; ANOVA: analysis of variance; AUC: area under the curve; BDI-II: Beck depression inventory version II; BMI: body mass index; BrdU: bromodeoxyuridine; ELISA: enzyme-linked immunosorbent assay; E:T: effector:target; HPA: hypothalamic-pituitary-adrenal; HRT: hormone replacement therapy; IL-6: interleukin-6; MUGA: multi-gated acquisition; NK: natural killer; PBMC: peripheral blood mononuclear cell; PHA: phytohemagglutinin; PSS: perceived stress scale; SIQR: semi-interquartile range; TNF-a: tumour necrosis factor-alpha.

## Competing interests

The authors declare that they have no competing interests.

## Authors’ contributions

JMS was the project lead, had a substantial involvement in the concept and design, analysis of biological samples, data analysis, interpretation, and drafting the manuscript; EJS was the main researcher responsible for the delivery of the intervention, acquisition of data (including analysis of biological samples) and revised the manuscript for intellectual content; AJD had a significant involvement in the overall conception and design, data interpretation and critical revision of the manuscript; MNW had a significant involvement in conception and design, including the choice of biological outcomes, acquisition of biological data and critical revision of the manuscript; NM was involved in the conception and design, data interpretation, critical revision of the manuscript; HC was involved in delivering the intervention, data acquisition and critical revision of the manuscript; HJP was involved in the conception and design, data interpretation, critical revision of the manuscript; REC guided recruitment, advised on clinical issues and was also involved in the conception and design, data interpretation and critical revision of the manuscript. All authors read and approved the final manuscript.
